# Supplementary figures and images for: Drosophila Myc restores immune homeostasis of Imd pathway via activating miR-277 to inhibit imd/Tab2
Source: PLoS Genet. 2020 Aug 18;16(8):e1008989. doi: 10.1371/journal.pgen.1008989 (PMC7455005; doi:10.1371/journal.pgen.1008989)

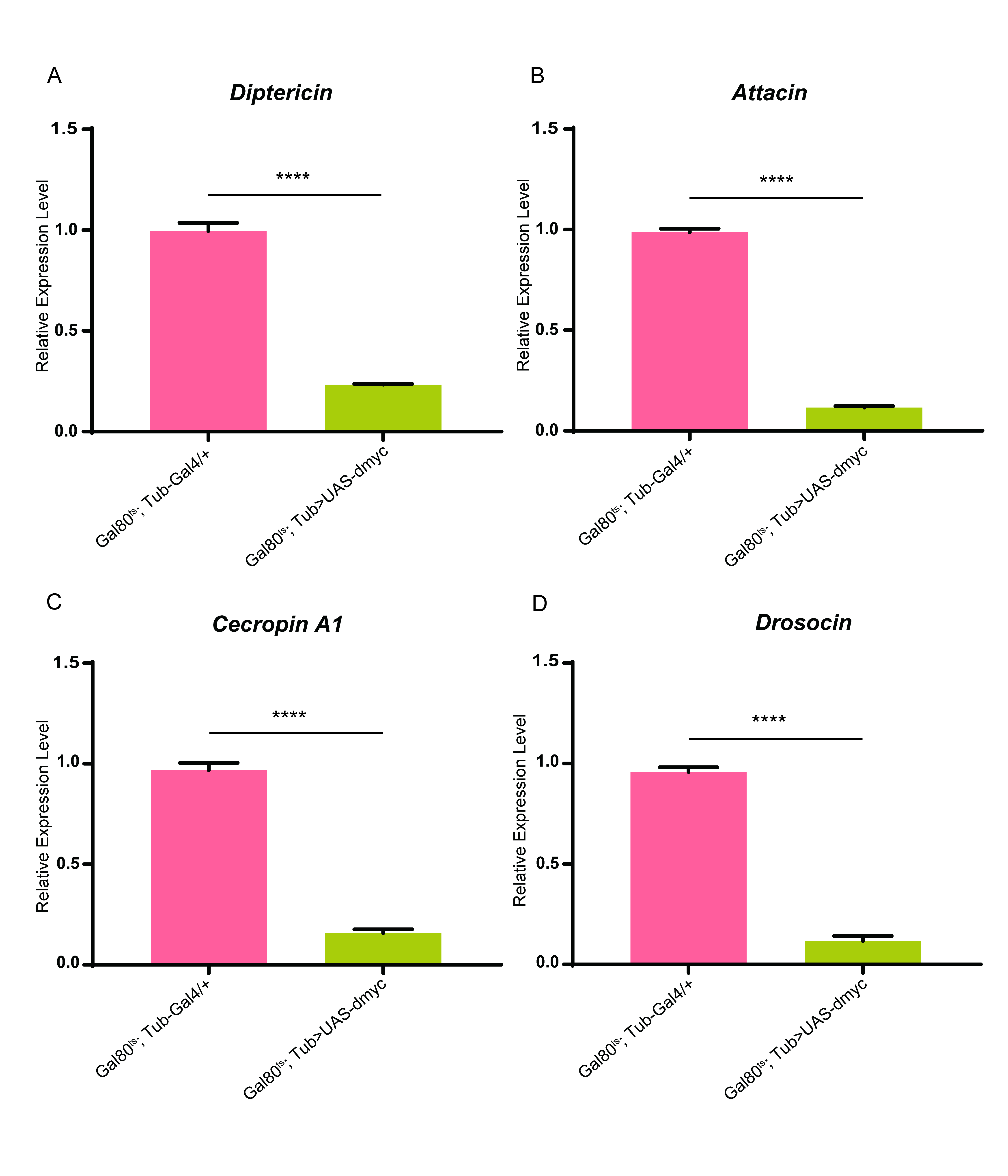

Supplement: S1 Fig — The expression level of Diptericin (A), Attacin (B), Cecropin A1 (C), and Drosocin (D) were measured in the dMyc high-expressing flies and the control flies upon E. coli infection. (TIF) [file pgen.1008989.s001.tif]

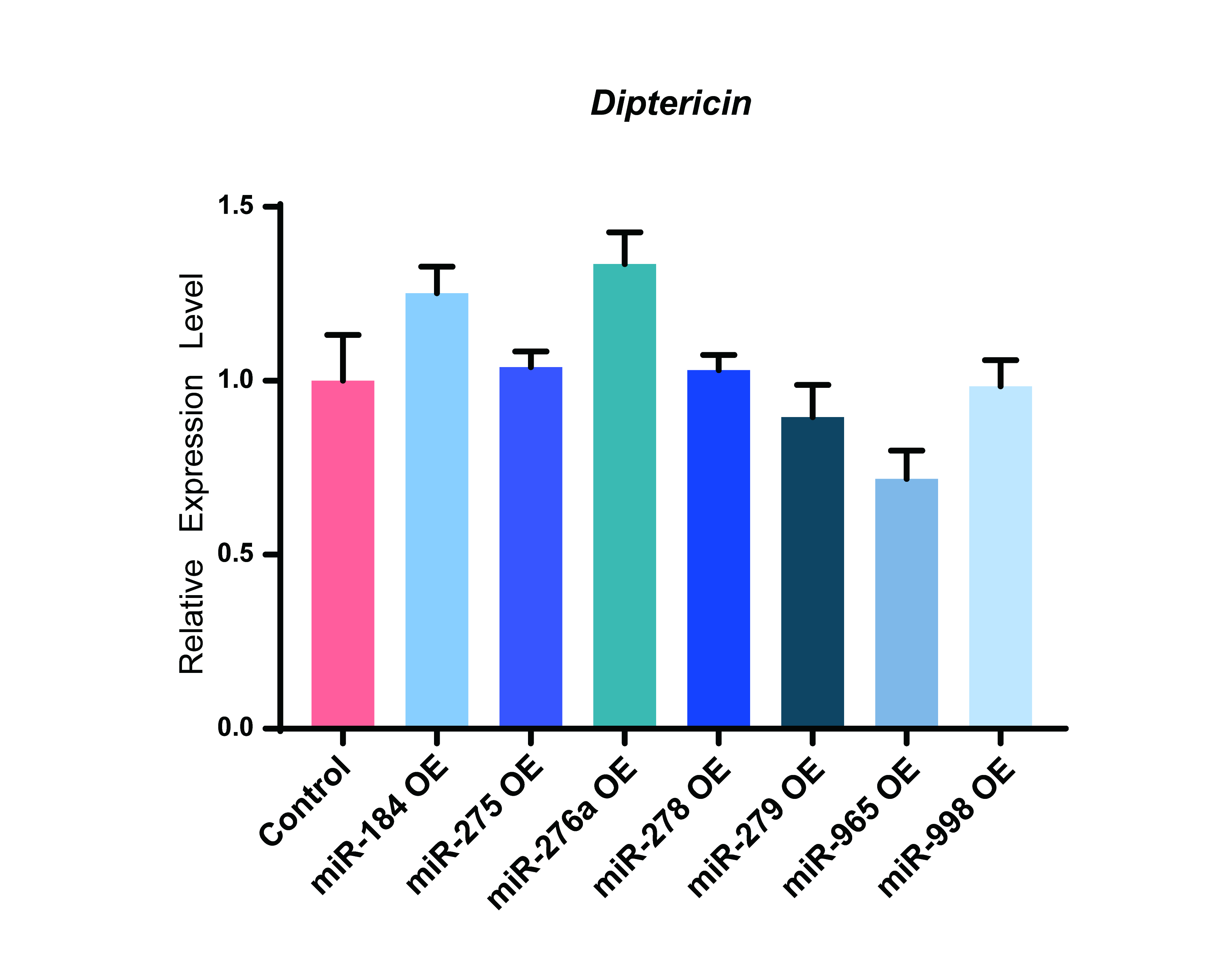

Supplement: S2 Fig — After 7 miRNAs were high-expressed respectively, the Dpt level was determined at 6 h upon E. coli infection. OE: overexpression. (TIF) [file pgen.1008989.s002.tif]

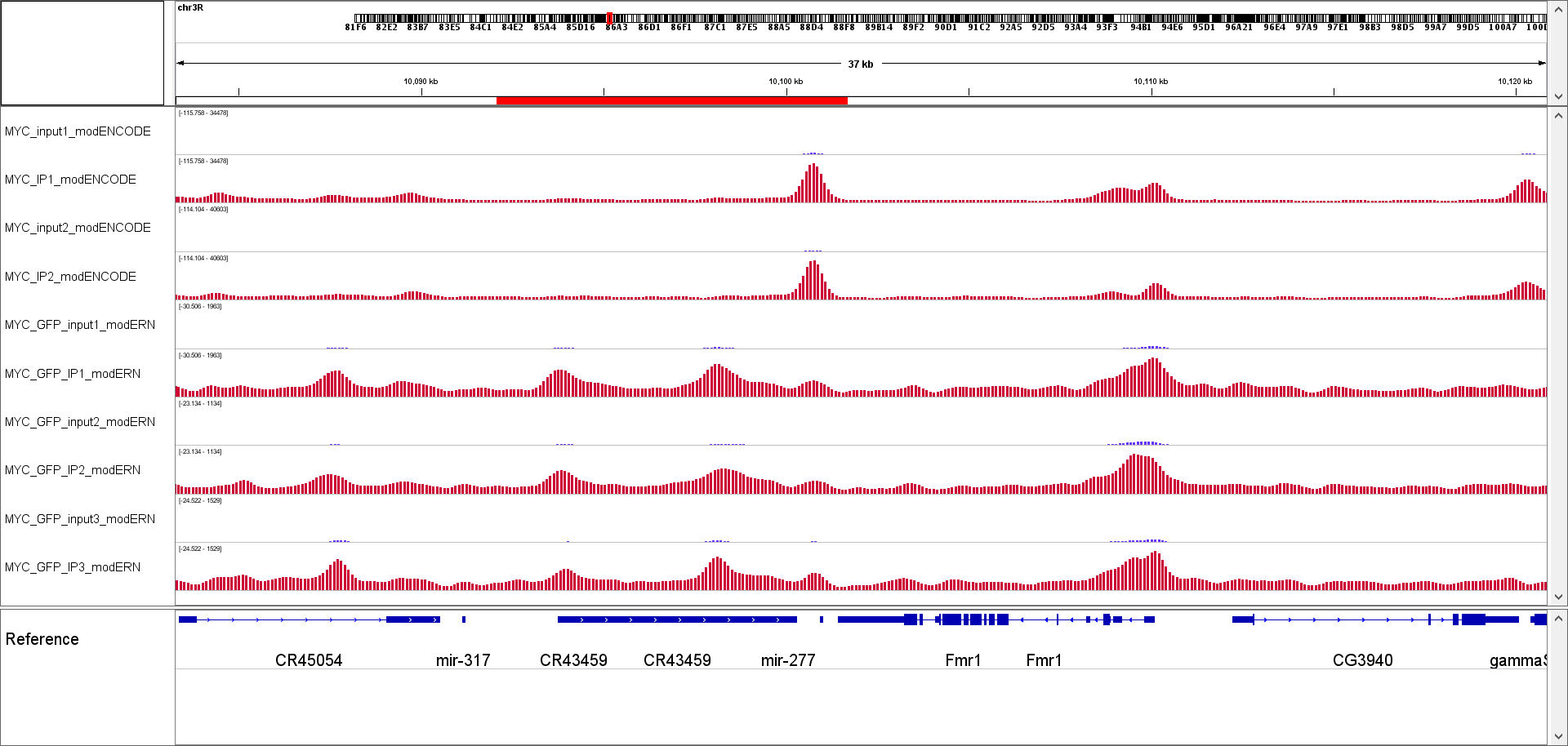

Supplement: S3 Fig — Two ChIP-seq data for dMyc from ENCODE database were visualized to show the bind sites of dMyc on the upstream of miR-277 gene. (TIF) [file pgen.1008989.s003.tif]

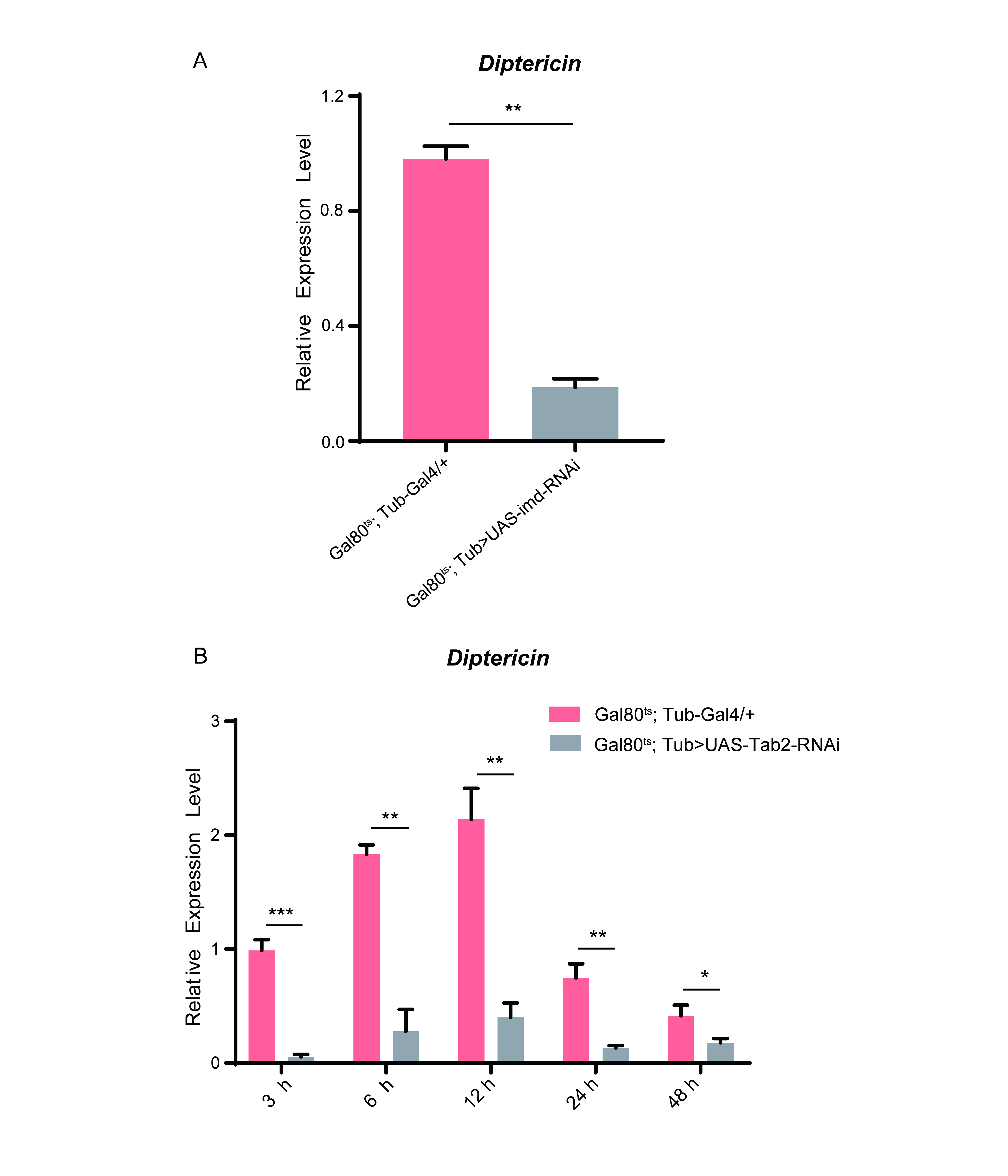

Supplement: S4 Fig — The expression level of Dpt was determined respectively by RT-q PCR in this imd-RNAi (A) and the Tab2-RNAi (B) highexpressed flies upon E. coli infection. (TIF) [file pgen.1008989.s004.tif]

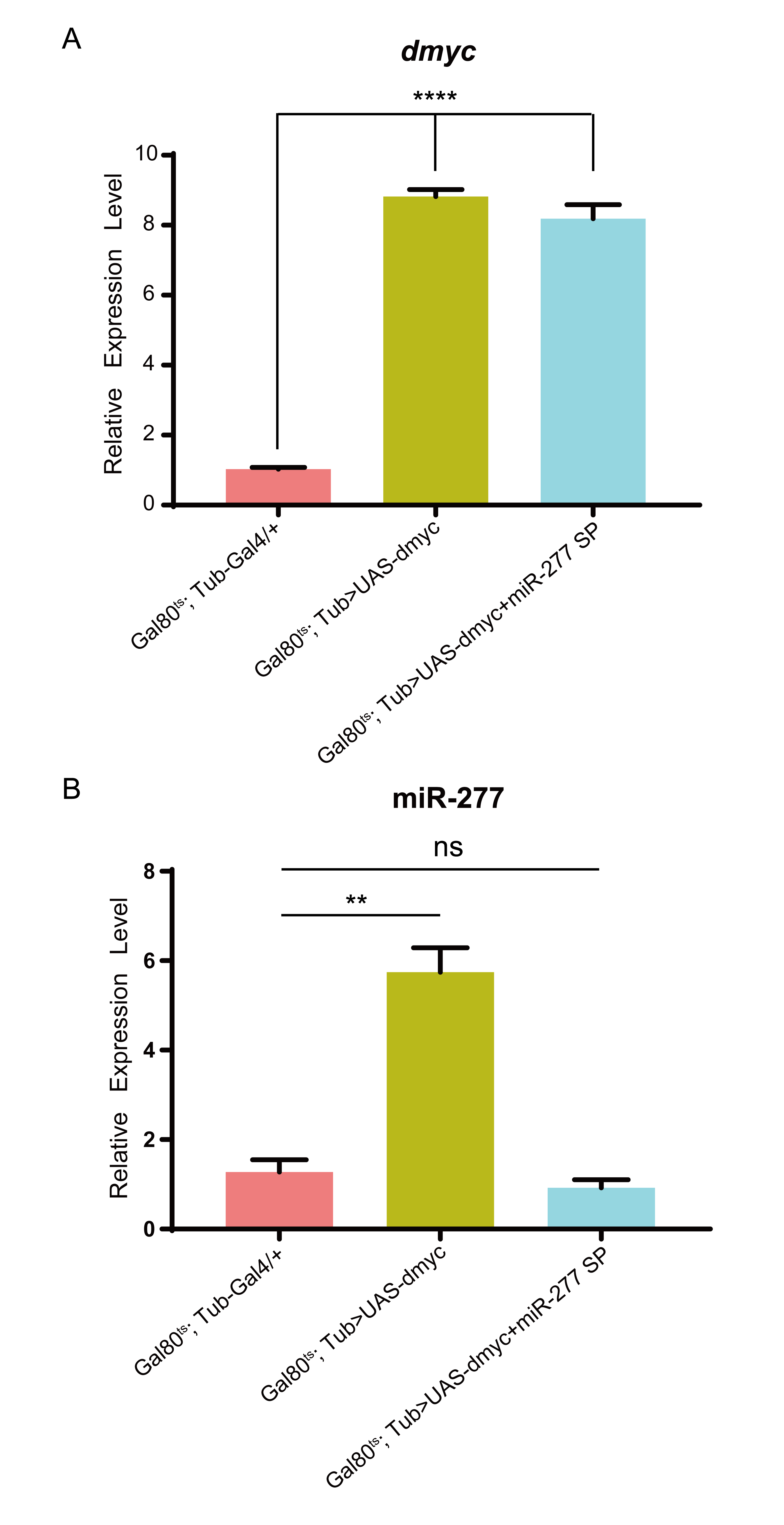

Supplement: S5 Fig — The expression levels of dMyc (A) and miR-277 (B) were examined in the control flies, the dMyc highexpressed flies, the dMyc and miR-277 sponge co-highexpressed flies before E. coli infection. (TIF) [file pgen.1008989.s005.tif]
