# Supplementary material for: Drosophila Myc restores immune homeostasis of Imd pathway via activating miR-277 to inhibit imd/Tab2
Source: PLoS Genet. 2020 Aug 18;16(8):e1008989. doi: 10.1371/journal.pgen.1008989 (PMC7455005; doi:10.1371/journal.pgen.1008989)
Supplement: S1 Table — (DOCX) [file pgen.1008989.s006.docx]

**Supplementary Table 1. Primers used for quantitative RT-PCR:**

| Name | Primer sequence |
| --- | --- |
| rp49-F | 5’- GACGCTTCAAGGGACAGTATCTG -3’ |
| rp49-R | 5’- AAACGCGGTTCTGCATGAG -3’ |
| Dpt-F | 5’- TCCGATGCCCGACGACATGA -3’ |
| Dpt-R | 5’- TGGCGTCCATTGTCGCTGGT -3’ |
| dmyc-F | 5’- GATATGGTGGACGATGGT -3’ |
| dmyc-R | 5’- CGGCAGATTGAAGTTATTGTA -3’ |
| imd-F | 5’- TTCGGCTCCGTCTACAACTT -3’ |
| imd-R | 5’- GTGATCGATTATGGCCTGGT -3’ |
| Tab2-F | 5’- TGTCATGGAGGAATGCGATC -3’ |
| Tab2-R | 5’- GCTTCTGACGCTCGATAGTGG -3’ |
| U6-F | 5’-CTTCGGCAGAACATATACTAA -3’ |
| U6-R | 5’-ATTTTGCGTGTCATCCTT -3’ |
| miR-277-RT | 5’-GTCGTATCCAGTGCAGGGTCCGAGGTATTCGCACTGGATACGACTGTCGT -3’ |
| miR-10-RT | 5’- GTCGTATCCAGTGCAGGGTCCGAGGTATTCGCACTGGATACGAC  AAACC -3’ |
| miR-1012-RT | 5’- GTCGTATCCAGTGCAGGGTCCGAGGTATTCGCACTGGATACGAC  TATTA -3’ |
| miR-2b-2-RT | 5’- GTCGTATCCAGTGCAGGGTCCGAGGTATTCGCACTGGATACGAC  GCTCC -3’ |
| miR-996-RT | 5’- GTCGTATCCAGTGCAGGGTCCGAGGTATTCGCACTGGATACGAC  AGACGA -3’ |
| miR-277-qF | 5’- CCGCTAAATGCACTATCTGG -3’ |
| miR-10-qF | 5’- GGCGCAAATTCGGTTCTAG -3’ |
| miR-1012-qF | 5’- CGCCTGGGTAGAACTTTGA -3’ |
| miR-2b-2-qF | 5’- GGCGTATCACAGCCAGCTT-3’ |
| miR-996-qF | 5’- GCGCTGACTAGATTTCATGC -3’ |
| miRNA-qR | 5’- CAGTGCAGGGTCCGAGGTAT -3’ |
