# Supplementary material for: Drosophila Myc restores immune homeostasis of Imd pathway via activating miR-277 to inhibit imd/Tab2
Source: PLoS Genet. 2020 Aug 18;16(8):e1008989. doi: 10.1371/journal.pgen.1008989 (PMC7455005; doi:10.1371/journal.pgen.1008989)
Supplement: S2 Table — (DOCX) [file pgen.1008989.s007.docx]

**Supplementary Table 2. Primers used for ChIP-qPCR:**

| Name | Primer sequence |
| --- | --- |
| Fibrillarin-F | 5’- TTTTACGCACCTGGTTTGCCCA -3’ |
| Fibrillarin -R | 5’- CCTCTCCGCCTGGTGTTGAACTT -3’ |
| ChIP1-F | 5’- GGAACCGAGCGATTTGACTG -3’ |
| ChIP1-R | 5’- CCCACATGCCCGTACATACA -3’ |
| ChIP2-F | 5’- CAGAAAACATTGGGAACT -3’ |
| ChIP2-R | 5’- GGCAGATAAGATAAGAAGG -3’ |
| ChIP3-F | 5’- GCAGATAATAAGTGGCAGAG -3’ |
| ChIP3-R | 5’- CGCTAAGACAACATACAAGT -3’ |
