# Supplementary material for: Drosophila Myc restores immune homeostasis of Imd pathway via activating miR-277 to inhibit imd/Tab2
Source: PLoS Genet. 2020 Aug 18;16(8):e1008989. doi: 10.1371/journal.pgen.1008989 (PMC7455005; doi:10.1371/journal.pgen.1008989)
Supplement: S3 Table — (DOCX) [file pgen.1008989.s008.docx]

**Supplementary Table 3. Primers used for transgene vector construction:**

| Name | Primer sequence |
| --- | --- |
| Myc CDS-flag-F | 5’- TGGTGGAATTCTGCAGATATCATGGATTACAAGGACGACGA  TGACAAGGCCCTTTACCGCTCTGAT -3’ |
| Myc CDS-R | 5’- GAAGGGCCCTCTAGACTCGAGCTATCCACTAACCGAGCGC  GA -3’ |
| P_TSS1_-F | 5’- GGTACCAACCGAGCGATTTGACTGG -3’ |
| P_TSS1_-R | 5’- AGATCT GTGGAGAGCAACGCAAAAC -3’ |
| P_TSS2_-F | 5’- GGTACCGCCCAATGCCTGCTCAATG -3’ |
| P_TSS2_-R | 5’- AGATCTGTGGGCTCCAAAAGGGTGT -3’ |
| miR-277-F | 5’- GAATTCGATAGACTGCCCCACA -3’ |
| miR-277-R | 5’- CTCGAGTTATCGCATTTTCCTG -3’ |
| imd 3’UTR-F | 5’- GAATTCTCAGTGAGTGAAACTTGCT -3’ |
| imd 3’UTR-R | 5’- CTCGAGCTGATTACGTAACAATAAAGCTTCT -3’ |
| Tab2-Ra/b 3’UTR-F | 5’- GAATTCCCCAAAAAGCTGCACCAA -3’ |
| Tab2-Ra/b 3’UTR-R | 5’- CTCGAGGTGTAAATATTTAAATAACGTTCT -3’ |
| Tab2-Rc 3’UTR-F | 5’- GATATCCAGTCGGCCAAACGCTCA -3’ |
| Tab2-Rc 3’UTR-R | 5’- CTCGAGCACGGTTTTCCATTACA -3’ |
