# Supplementary material for: Drosophila Myc restores immune homeostasis of Imd pathway via activating miR-277 to inhibit imd/Tab2
Source: PLoS Genet. 2020 Aug 18;16(8):e1008989. doi: 10.1371/journal.pgen.1008989 (PMC7455005; doi:10.1371/journal.pgen.1008989)
Supplement: Attachment — Submitted filename: Response to reviewers.docx [file pgen.1008989.s009.docx]

**Dear Editor:**

Thanks for your kind consideration and comments regarding to our manuscript entitled “*Drosophila* Myc restores immune homeostasis of Imd pathway via activating miR-277 to inhibit imd/Tab2” (PGENETICS-D-19-02099).

We have revised our manuscript (details are provided below) taken into account of all comments and suggestions from the reviewers. In addition, we have made extensive modifications for English grammar in this revised manuscript. We wish the revised manuscript can meet the standard requirement for *PLOS Genetics*. We look forward to your favorable feedbacks.

Thanks a lot for everything.

Sincerely yours,

Fei Ma

College of Life Sciences

Nanjing Normal University

Nanjing 210023

P. R. China

E-mail: mafei01@tsinghua.org.cn

Fax: +86-25-85891050

**Response to reviewer1’s comments:**

**Reviewer #1:** In this manuscript, Li et al study the link between IMD pathway, Myc and miR-277. They propose that Myc represses the IMD pathway and that this is mediated by the miR-277. There are many problems in this manuscript that are listed here.

**Comment 1:** All the presented experiments analyzed the effects of Myc over expression using the Gal4 system. So, they do not study the role of Myc in the immune homeostasis but the consequences of Myc overexpression. This is therefore non-physiological and might have nothing to do with the endogenous role of Myc. Loss-of-function mutants should be use to study the role of a gene in a given process.

**Response:** Thank you for your constructive comments. As you suggested, the physiological function of dMyc in the IMD pathway was further carried out in wild-type flies. We explored the dynamic expressions of *Dpt*, *dMyc*, miR-277, *imd* and *Tab2* in wild-type flies at 0, 3, 6, 12, 24 and 48 h after *E. coli* infection. This results showed the relationship between dMyc and IMD-related factors (Fig.1 for the Reviewer), which consistently supported the physiological function of dMyc in the IMD pathway and immune homeostasis. This part works have been added in Fig 7 of our revised manuscript. Thus, we suggested that dMyc can restore IMD immune homeostasis based on the results of overexpression and Loss-of-function as well as the dynamic changes of five genes.

**Fig 1. The temporal expression patterns of four genes and** **miR-277 in the wild-type flies prior to and following *E.coli* infection.** The dynamic expression changes of *Dpt* (A), *dmyc* (B), miR-277 (C), *imd* and *Tab2* (D) at six time-points (0, 3, 6, 12, 24 and 48 h) prior to and following *E. coli* infection, respectively.

**Comment 2:** Most of the experiments presented here use the diptericin as a read out of the IMD pathway. Hundreds of papers have shown that, as most Antimicrobial peptides, diptericin transcripts can increase us to 10.000 times upon infection. Even in the absence of infection, their levels vary quite a bit. The fold changes reported in this manuscript are around 1,5 or even sometimes less. There is something wrong here. I do not what. When a gene can have its transcript produced 10.000 times more in control conditions, what does a 1,3 fold difference mean? My guess is that there is a technical problem.

**Response:** Thank you very much for your comment. As the reviewer mentioned, many studies have indicated that *diptericin* transcript can increase to 100-1000 times after infection [1-3]. To avoid potential confusions, in our revised manuscript, we repeated our experiments via adding the 0 h control group as the basis for normalization to all experiments. Our results showed that the fold changes of *diptericin* transcripts are around 400 times upon infection than before infection (Fig.2, Fig.3 and Fig.4 for the Reviewer). We have added these results in Fig.1, Fig.4 and Fig.6 in this revised manuscript, respectively.

**Fig.2 The expression level of *Diptericin* in dMyc mutant flies before and after infection with *E. coli***

(A) The expression level of Diptericin was measured by qRT-PCR in dMyc high-expressing flies before and after infection with *E.coli*. (B) The expression level of *Diptericin* was measured by qRT-PCR in the other dMyc mutant flies in the absence of infection. (C) The expression level of *Diptericin* was measured by qRT-PCR in the other dMyc mutant flies after infection with *E.coli* for 6 h.

**Fig.3 The expression level of *Diptericin* in miR-277 mutant flies before and after infection with *E. coli.*** (A) The expression level of Diptericin was measured by qRT-PCR in miR-277 high-expressing flies before and after infection with *E.coli*. (B) The expression level of *Diptericin* was measured by qRT-PCR in the miR-277 and miR-277 sponge co-highexpressing flies in the absence of infection. (C) The expression level of *Diptericin* was measured by qRT-PCR in the miR-277 and miR-277 sponge co-highexpressing flies after infection with *E.coli* for 6 h.

**Fig.4 The expression level of *Diptericin* in dMyc and miR-277 sponge co-highexpressing flies before and after infection with *E. coli.*** (A) The expression level of *Diptericin* was measured by qRT-PCR in the dMyc and miR-277 sponge co-highexpressing in the absence of infection. (B) The expression level of *Diptericin* was measured by qRT-PCR in the dMyc and miR-277 sponge co-highexpressing flies after infection with *E.coli* for 6 h.

**Comment 3:** The experiments always miss important controls. Just one example but this is true for all of them. Fig 8, the authors test the resistance of Myc overexpressing flies to *E. Cloacae* infection. The survival rate of these flies in the absence of infection and after sterile wounding should be tested.

**Response:** Thank you for your valuable suggests. Based on your recommendation, we further detected the expression level of *Diptericin* in all mutant flies in the absence of infection in this revised manuscript, finding that there was no significant differences in the expression level of *Diptericin* within all mutant flies without infection (Fig.5 for the Reviewer), and these results have been added in Fig1C, Fig 4C and Fig 6A of this revised manuscript, respectively.

**Fig.5 The expression level of *Diptericin* in all mutant flies in the absence of infection.** (A) The expression level of *Diptericin* was measured by qRT-PCR in the dMyc mutant flies in the absence of infection. (B) The expression level of *Diptericin* was measured by qRT-PCR in the miR-277 mutant flies in the absence of infection. (C) The expression level of *Diptericin* was measured by qRT-PCR in the dMyc and miR-277 sponge co-highexpressing flies in the absence of infection.

At the suggestion of you, in this revised manuscript, we further investigated the survival rate of dMyc high-expressed flies and the control flies (Gal80^ts^; Tub-Gal4/+) without infection and with PBS as well as *E. cloacae* infection, respectively. Our result demonstrated that the survival rate of dMyc overexpressed flies was significantly lower than the control groups in the absence of infection and after infection with PBS, whilst the overall survival rate of dMyc high-expressed flies was significantly higher than the control groups after infection with *E. cloacae*. These results have been added in the Fig.8 of this revised manuscript.

**Comment 4:** Immune genes can be activated by stress. Overexpressing the oncogene Myc in a cell is probably stressful for the flies. I am not sure this work studies immune response but rather stress response.

**Response:** As you mentioned, we agree that immune genes can be activated by stress. To address this point, we examined the expression level of *Diptericin* in dMyc overexpressing flies in the absence of infection. The results showed that there was no significant difference in the expression level of *Diptericin* between the dMyc overexpressing fly without infection and the control group (the above Fig.5 for the Reviewer ). Remarkably, as shown in Fig.1A of this revised manuscript, after dMyc high-expressed flies with *E. coli* infection, the expression level of *Diptericin* in dMyc high-expressed flies was significantly lower than the control groups, instead of upregulating the response of the IMD pathway. Taken together, These results indicate that potential stress via overexpressing dMyc has negligible effects on regulation of immune genes compared to infection.

**Comment 5:** The text is very difficult to follow. Just one example from the first sentence of the results “as we know that the diptericin expression is an important readout of Drosophila Imd pathway activation”. We expect the phrase to continue but it does not.

**Response:** Thank you for your suggestion. We have made a careful and extensive revision of the contents in this revised manuscript.

**Reference:**

1.	Zhang L, Xu W, Gao X, Li W, Qi S, Guo D, et al. lncRNA Sensing of a Viral Suppressor of RNAi Activates Non-canonical Innate Immune Signaling in Drosophila. Cell host & microbe. 2020;27(1):115-28 e8. doi: 10.1016/j.chom.2019.12.006. PubMed PMID: 31917956.

2.	Valanne S, Salminen TS, Jarvela-Stolting M, Vesala L, Ramet M. Immune-inducible non-coding RNA molecule lincRNA-IBIN connects immunity and metabolism in Drosophila melanogaster. PLoS Pathog. 2019;15(1):e1007504. doi: 10.1371/journal.ppat.1007504. PubMed PMID: 30633769; PubMed Central PMCID: PMC6345493.

3.	Xiong XP, Kurthkoti K, Chang KY, Li JL, Ren X, Ni JQ, et al. miR-34 Modulates Innate Immunity and Ecdysone Signaling in Drosophila. PLoS Pathog. 2016;12(11):e1006034. doi: 10.1371/journal.ppat.1006034. PubMed PMID: 27893816; PubMed Central PMCID: PMC5125713.

**Response to reviewer2’s comments:**

**Reviewer #2:** The manuscript “Drosophila Myc restores immune homeostasis of Imd pathway via activating miR-277 to inhibit imd/Tab2” (PGENETICS-D-19-02099) has revealed that Drosophila transcription factor dMyc could serve as a novel negative regulator of Imd pathway immune response using the loss- and gain-of-function screening. Especially, their works illuminate a feasible mechanism that dMyc as a transcription factor may activate miR-277 transcription to inhibit imd and/or Tab2 expression to restore immune homeostasis of Drosophila Imd pathway and improve the survival of flies. Overall, this manuscript has provided new insights into further studying the maintenance mechanism of innate immune homeostasis. The content of this manuscript is interesting. Thus I think that this manuscript is suitable to be published in PLOS Genetics. However, there are several shortcomings as following:

**Major comments**

**Comment 1:** In response to Gram-negative bacteria, the Imd pathway produces not only Dpt but also other antimicrobial peptides (AMPs). Thus, what are the expression levels of other AMPs in response to E.coli?

**Response:** As the reviewer said, besides Dpt, in Imd pathway, there are other the immune-inducible AMPs. We further detected the expression level of other AMPs, including *Drosocin*, *Attacin* and *Cecropin A1.* As shown in Fig.1 and Fig.2 below, their expression levels were similar to *Dpt* in the miR-277 or/and dMyc mutant flies. Thus, we chose the *Dpt* as the representative for exploring Imd immune response in this work.

**Fig.1 The expression level of multiple AMPs in the miR-277 mutant flies.** The expression level of *Diptericin* (A), *Attacin* (B), *Cecropin A1* (C), and *Drosocin* (D) were measured in miR-277 high-expressing flies and miR-277 and miR-277 sponge co-highexpressing flies upon *E. coli* infection.

**Fig.2 The expression level of multiple AMPs in the dMyc and miR-277 mutant flies.** The expression level of *Diptericin* (A), *Attacin* (B), *Cecropin A1* (C), and *Drosocin* (D) were measured in dMyc high-expressing flies and dMyc and miR-277 sponge co-highexpressing flies upon *E. coli* infection.

**Comment 2:** Although the expression level of dMyc in the dMyc and dMyc-RNAi co-highexpressed flies could be recovered to some extent, whether the expression level of miR-277 could also be restores?

**Response:** Thank you for your valuable comment. To address this, we tested the expression level of miR-277 in the dMyc and dMyc-RNAi co-highexpressed flies. The result shows that there was no significant difference in the expression level of miR-277 between the dMyc and dMyc-RNAi co-highexpressed flies and the control group (Fig.3), indicating that the expression level of miR-277 can be restored.

**Fig.3 The expression level of miR-277 in the dMyc and dMyc-RNAico-highexpressing flies.** The qRT-PCR analysis was performed in dMyc and dMyc-RNAi co-highexpressing flies upon *E. coli* infection to detect the expression level of miR-277.

**Comment 3:** Myc may recognize some binding motifs so-called E-boxes (e.g. CACGTG, CATGTG and alternative sequences) in the promoter region of target genes to activate their expression. Whether there is a similar E-box in the promoter region of miR-277? Especially, when the E-box of miR-277 is deleted or mutated, whether Myc could still activate the transcription of miR-277？

**Response:** To address these points, first, we searched for the binding motifs of dMyc in the promoter sequence of miR-277 and found that there is the “CACGTG” motif in the upstream vicinity of TSS1. After the “CACGTG” motif was deleted, the luciferase activity was significantly down-regulated (Fig.4).

**Fig.4 The** **luciferase activity analysis of the mutant promoter sequence of miR-277.** After the E-box motif in the promoter sequence of miR-277 was deleted, the promoter activity was analyzed.

**Comment 4:** Why choose these five time points to collect and test samples after infection?

**Response:** We chose these five time-points (3, 6, 12, 24, 48 h) to collect and test samples after infection, because in our previously studies, we have show that these five time-points represented the whole process of the immune response, including the initiation, persistence, peak, and recovery to normal level [1].

**Minor comments**

**Comment 1:** Some abbreviations, such as E.coli and LPS, should write the full name at the first appearance.

**Response:** We have revised these points accordingly in the revised manuscript.

**Comment 2:** In Fig 3, the font of the title is inconsistent.

**Response:** We have revised this issue in the revised manuscript.

**Comment 3:** In Fig 7, line 326: “E.col” should be “E.coli”.

**Response:** Sorry for the typo. We have corrected this point in the revised manuscript.

**Comment 4:** Line 430: “togther” should be “together”.

**Response:** Sorry for the typo. We have corrected this point in the revised manuscript.

**Comment 5:** “(D)”should be “(B)” in Fig 1 legend (line 140).

**Response:** We have corrected this point in the revised manuscript.

**Comment 6:** “Drosophila” should be italic (line 116 and 148).

**Response:** We have corrected this point in the revised manuscript.

**Comment 7:** The legend in Fig4A is not consistent with other figures. It should be “Gal80ts” not “Gal80”.

**Response:** Sorry for the typo. We have corrected this point in the revised manuscript.

**Comment 8:** Two legends in Fig 3B and 3C could look better when they are adjusted up and down.

**Response:** Thank you for your suggestion. We have made an adjustment in the revised manuscript.

**Reference:**

1. Li S, Shen L, Sun L, Xu J, Jin P, Chen L, et al. Small RNA-Seq analysis reveals microRNA-regulation of the Imd pathway during Escherichia coli infection in Drosophila. Developmental and comparative immunology. 2017;70:80-7. doi: 10.1016/j.dci.2017.01.008. PubMed PMID: 28069431.

**Response to reviewer3’s comments:**

**Reviewer #3:** Li et al. have studied the effect of Drosophila Myc on innate immune signaling. They have discovered that it acts as a negative regulator of the IMD pathway and they further show that it acts by inducing expression of miR-277 transcription which in turn down-regulates the expression of imd and Tab2-Ra/b (but not Tab2-Rc). Notably, dMyc was shown to bind to the promoter region of miR-277.

**Major comments:**

Most of all, the level of the English grammar – and the way of scientific writing – is not at the required level. As I see potential in this manuscript I hope that there would be careful editing for the paper for scientific English.

**General Response**: Thank you for your comment. We had made the extensive modifications to correct the English grammar in the revised manuscript.

**Comment 1:** Figure 1 A, Dipt expression should be shown also in uninfected (0h) flies.

**Response:** Thank you for your suggestion. In our revised manuscript, we repeated our experiments via adding the 0 h control group. As shown in Fig.1 below, there was no significant differences in the expression level of *Diptericin* within all mutant flies without infection. These results have been added in Fig1C, Fig 4C and Fig 6A of this revised manuscript, respectively.

**Fig.1 The expression level of *Diptericin* in all mutant flies in the absence of infection.** (A) The expression level of *Diptericin* was measured by qRT-PCR in the dMyc mutant flies in the absence of infection. (B) The expression level of *Diptericin* was measured by qRT-PCR in the miR-277 mutant flies in the absence of infection. (C) The expression level of *Diptericin* was measured by qRT-PCR in the dMyc and miR-277 sponge co-highexpressing flies in the absence of infection.

**Comment 2:** Figure 3B: S2 cells respond poorly to LPS as in Drosophila, gram-negative bacteria are recognized by Peptidoglycan recognition proteins, predominantly via PGRP-LC (use Choe et al., 2002 Nature, Ramet et al., 2002 Nature, Gottar et al., 2002 Science for references). I suggest repeating this experiments using heat-killed *E. coli* instead of LPS.

**Response:** Thank you for your good suggestion. We agree that gram-negative bacteria can be recognized by Peptidoglycan recognition proteins, predominantly via PGRP-LC. In fact, several previous studies have shown that highly purified LPS could not induce the expression of antibacterial peptide genes in *Drosophila* [1]. However, other studies have demonstrated that the commercial LPS from *E. coli* 055:B5 (Sigma, St. Louis, MO) can well trigger the immune response of Imd pathway in *Drosophila* S2 cells [2]. Our results are also consistent with these above reports (Fig.2 below). We speculated that this commercial LPS may contain a peptidoglycan activating component. In addition, as you suggested, we also attempted to use heat-killed *E. coli* instead of LPS. However, our results indicated that the Imd pathway in *Drosophila* S2 cells can be weakly activated, and the expression level of *Diptericin* is only about three times higher than the control (Fig.3 below). Here, we are very sorry that we do not use heat-killed *E. coli* instead of LPS to further detect immune response of Imd pathway in *Drosophila* S2 cells.

**Fig.2 The expression level of *Diptericin* in *Drosophila* S2 cells stimulated by commercial LPS.** The expression level of *Diptericin* was measured by qRT-PCR in *Drosophila* S2 cells stimulated by this commercial LPS.

**Fig.3 The expression level of *Diptericin* in *Drosophila* S2 cells stimulated by** **heat-killed *E. coli*.** The expression level of *Diptericin* was measured by qRT-PCR in *Drosophila* S2 cells stimulated by heat-killed *E. coli*.

**Minor comments:**

**Comment 1**: Lines 75-77 ‘Furthermore, the Imd-mediated immune response can also be negatively regulated by some immune suppressors, such as WntD, Die, PGRP-LF, pirk, dUSP36, CYLD, Dnr1, dRYBP and Caspar [15-24]’ lacks the notion and reference to the most important negative regulator of this pathway, namely Pirk (references Kallio et al., 2005 Microbes and Infection and Kleino et al., J Immunol 2008 needs to be cited).

**Response:** Thank you for your good suggestion. We have updated these references in this revised manuscript.

**Comment 2**: Line 117: ‘As we all know…’ Rephrase

**Response:** Thank you for your suggestion. We have rephrased the sentence in this revised manuscript.

**Comment 3**: Lines 124 Avoid phrases such as ‘remarkably’ – the results will speak to themselves

**Response:** We have made a modification in this revised manuscript.

**Comment 4**: Lines 128-129: ‘Especially, the expression level of Diptericin in the dMyc and dMyc-RNAi co-highexpressed flies’ – I see no point – is I understand this correctly – to both overexpress dMyc and to suppress its expression by RNAi at the same time.

**Response:** We intended to restore dMyc expression to the normal level in the context of high-expressing dMyc through introducing dMyc-RNAi to mimic the physiological condition.

**Comment 5:** Fig 1C: I see no reason to cut the y-axis

**Response:** Thank you for your comment. We have revised this point in this revised manuscript.

**Comment 6:** Line 189: ‘This dMyc can activate…’ Remove word ‘This’

**Response:** Thank you for your comment. We have removed the word ‘This’ in this revised manuscript.

**Comment 7:** Figure 3 B and C – statistical significance

**Response:** Thank you for your good suggestion. We have performed a statistical analysis and added the statistical results to the Figure 3 B and C in this revised manuscript.

**Comment 8:** Chapter ‘miR-277 inhibits the expression of imd and Tab2-Ra/b’ – Authors should –shortly introduce why they are interested in Tab2, i.e. that it has been shown to be a key component of the Drosophila Imd pathway (Kleino et al., 2005 EMBO J)

**Response:** Thank you for your good suggestion. We have updated this reference in this revised manuscript.

**Comment 9:** Fig 7C – there is a mistake in the y-axis (spelling)

**Response:** We have corrected this in the revised manuscript.

**Comment 10:** Line 326 - E.col infection

**Response:** Sorry for the typo. We have corrected “E.col infection” to “E. coli infection” in this revised manuscript.

**Comment 11:** Figure 8 – there is no reason to cut the x-axis

**Response:** Thank you for your comment. We have revised this point in fig. 8 in this revised manuscript.

**Comment 12:** Line 451: ‘And that dMyc…’ Rephrase

**Response:** We have rephrased the sentence in the revised manuscript.

**Reference:**

1.	Kaneko T, Goldman WE, Mellroth P, Steiner H, Fukase K, Kusumoto S, et al. Monomeric and polymeric gram-negative peptidoglycan but not purified LPS stimulate the Drosophila IMD pathway. Immunity. 2004;20(5):637-49. doi: 10.1016/s1074-7613(04)00104-9. PubMed PMID: 15142531.

2.	Leulier F, Parquet C, Pili-Floury S, Ryu JH, Caroff M, Lee WJ, et al. The Drosophila immune system detects bacteria through specific peptidoglycan recognition. Nat Immunol. 2003;4(5):478-84. doi: 10.1038/ni922. PubMed PMID: 12692550.
